# Developmental deltamethrin: Sex-specific hippocampal effects in Sprague Dawley rats

**DOI:** 10.1016/j.crtox.2022.100093

**Published:** 2022-11-02

**Authors:** Emily M. Pitzer, Chiho Sugimoto, Samantha L. Regan, Gary A. Gudelsky, Michael T. Williams, Charles V. Vorhees

**Affiliations:** a Dept. of Pediatrics, University of Cincinnati College of Medicine, and Division of Neurology, Cincinnati Children’s Hospital Medical Center, Cincinnati, OH 45229, USA; b Dept. of Physiology, Michigan State University, 766 Service Rd. 5401 Interdisciplinary Science and Technology Building, East Lansing, MI 48824, USA; c Dept. of Human Genetics, University of Michigan Medical Center, 3703 Med Sci II, 1241 E. Catherine St., Ann Arbor, MI 48109-5618, USA; d College of Pharmacy, Div. of Pharmaceutical Sciences, 3212 Medical Sciences Building, University of Cincinnati, Cincinnati, OH 45219, USA

**Keywords:** Deltamethrin, Glutamate, Dopamine, Long-term potentiation, Pyrethroids, Rats, Caspase-3, Apoptosis, Cytokines, DA, dopamine, LTP, Long-term potentiation, DLM, deltamethrin

## Abstract

Pyrethroid pesticides are widely used and can cause long-term effects after early exposure. Epidemiological and animal studies reveal associations between pyrethroid exposure and altered cognition following prenatal and/or neonatal exposure. However, little is known about the cellular effects of such exposure. Sprague Dawley rats were gavaged with 0 or 1.0 mg/kg deltamethrin (DLM), a Type II pyrethroid, in corn oil (dose volume 5 mL/kg) once per day from postnatal day (P) 3–20 and assessed shortly after dosing ended or as adults. No effects of DLM exposure were found on striatal dopaminergic markers, nor on AMPA receptor subunits or on NMDA-NR1. However, DLM increased NMDA-NR2A and decreased NMDA-NR2B levels in the hippocampus, in males but not females. Additionally, adult hippocampal CA1 long-term potentiation was increased in DLM-treated males but not females. Potassium stimulated extracellular glutamate release in the hippocampus was not affected using *in vivo* microdialysis. Terminal deoxynucleotidyl transferase dUTP nick end labeling (TUNEL) showed increased apoptotic cells in the dentate gyrus of male rats, in the absence of changes in cleaved caspase-3 at P21. Proinflammatory cytokines interferon gamma trended up in striatum, interleukin-1β trended down in nucleus accumbens, IL-13 trended up in hippocampus, and keratinocyte chemoattractant/human growth-regulated oncogene (KC/GRO or CXCL1) was significantly increased in the hippocampus in male DLM-treated rats on P20. The data point to the developing hippocampus as a susceptible region to DLM-induced adverse effects.

## Highlights

• Low dose deltamethrin (DLM) effects have been reported in developing rodents.
• Rats treated with DLM from P3-20 showed effects on NMDA-NR2A and NMDA-NR2B.
• DLM increased apoptosis in the dentate gyrus by TUNEL staining.
• DLM altered hippocampal long-term potentiation (LTP).
• DLM has multiple long-term effects on rat brain after early exposure.

## Graphical abstract

**Figure:**
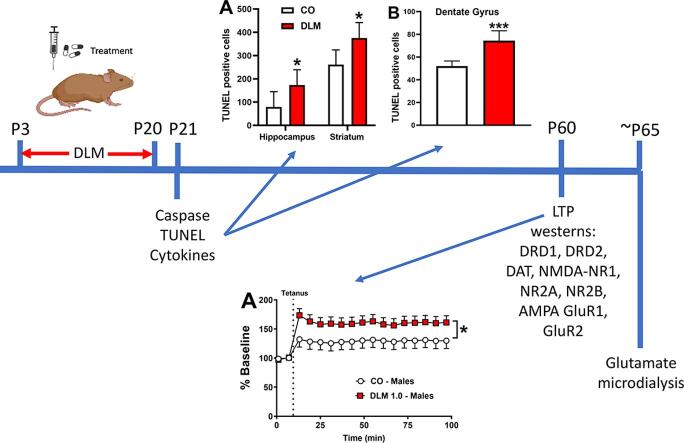

## 1 Introduction

Deltamethrin (DLM) is a type II pyrethroid pesticide used to kill ectoparasites on animals, insects on agricultural crops, and in public health programs to kill mosquitos for the control of malaria and other disease carrying insects. Other applications include use to control insects on lawns, playgrounds, parks, houses, apartments, schools, and businesses. They are used on pets to kill ticks and fleas and on children to treat head lice. Pyrethroid use has increased because of the phase-out of more toxic pesticides (Barr et al., 2010, Saillenfait et al., 2015).

Adults metabolize pyrethroids efficiently with plasma half-lives of ∼11.5 h (Chrustek et al., 2018). Children interact with treated surfaces more than adults yet metabolize these chemicals slower. They also have less mature blood brain barriers (BBB) than adults (Lu et al., 2006, Lu et al., 2010, Morgan, 2012). Epidemiological studies find correlations for urinary 3-phenoxybenzoic acid (3-PBA) levels, a metabolite of several pyrethroids, and neurobehavioral disorders that include autism spectrum disorder (ASD), attention deficit hyperactivity disorder (ADHD), and developmental delay (Oulhote and Bouchard, 2013, Xue et al., 2013, Shelton et al., 2014, Richardson et al., 2015, Viel et al., 2015, Wagner-Schuman et al., 2015). Although concerns have been raised about these studies (Burns and Pastoor, 2018), the implications about the safety of pyrethroids during brain development merit further investigation.

In this regard, data from animal experiments add to concerns about pyrethroid effects during development. For example, immature rats are more susceptible to overt signs of pyrethroid neurotoxicity than adult rats (Cantalamessa, 1993, Sheets et al., 1994, Williams et al., 2019). Physiologically based pharmacokinetic (PBPK) models show that plasma and brain DLM levels are inversely related to age, i.e., higher concentrations of DLM remain longer in the brain and plasma of preweaning rats as compared with adult rats given the same dosage (Kim et al., 2010). This is because young rats have less capacity to metabolize these compounds (Anand et al., 2006) combined with an increased BBB permeability (Amaraneni et al., 2017).

We previously found deficits in learning and memory following postnatal day (P) 3–20 DLM exposure in Sprague Dawley rats (Pitzer et al., 2019). DLM-treated rats showed decreased locomotor activity, increased acoustic startle, and increased hippocampal long-term potentiation (LTP). Additionally, DLM-treated rats were impaired for egocentric navigation, reduced spatial reversal learning, altered contextual conditioned freezing, and attenuated locomotor activity to the NMDA antagonist MK-801. Several of these effects were male-specific. Studies in mice and rats from others have also reported developmental DLM effects on behavior (Aziz et al., 2001, Johri et al., 2006, Richardson et al., 2015).

Developmental DLM exposure affects several dopamine (DA) markers (Lazarini et al., 2001, Pitzer et al., 2019). We previously found that male DLM-treated offspring had decreased DA D1 receptor (*Drd1*) mRNA in the neostriatum and decreased stimulated DA release in the nucleus accumbens by microdialysis compared with controls (Pitzer et al., 2019). In mice, DLM-treatment (3 mg/kg every 3 days, E0-P21) resulted in decreased DA release in the nucleus accumbens, as well as increased DA transporter (DAT) and DRD1 protein levels (Richardson et al., 2015). Lazarini et al. (2001) found that DLM exposure (E6-15; 0.08 mg/kg, by gavage) in rats resulted in increased 3,4-dihydroxyphenylacetic acid (DOPAC) and DOPAC/DA ratios compared with controls (Lazarini et al., 2001).

Adult animals have had effects from DLM treatment at higher doses and in cell culture where it causesd endoplasmic reticulum (ER) stress resulting in cell death in SK-*N*-AS cells (Hossain and Richardson, 2011) as well as *in vivo* in mice (Hossain et al., 2015). DLM activates inflammatory and immune pathways that trigger cell apoptotic reactions (Hossain et al., 2017). In addition, DLM activates microglia when it binds to voltage gated sodium channels (Black et al., 2009, Stevens et al., 2013, Pappalardo et al., 2016, Hossain et al., 2017), resulting in cytokine release (Black et al., 2009, Morsali et al., 2013, Stevens et al., 2013, Pappalardo et al., 2016).

Here we kept the exposure period, dose, carrier, and dose volume the same as we used previously (P3-20 DLM) (Pitzer et al., 2019), and assessed whether the LTP changes we found at P25-35 persisted into adulthood. We assessed DA markers associated with egocentric navigation, that is striatum and nucleus accumbens mediated. Since reversal spatial learning was affected in the Morris water maze (MWM), we assessed glutamatergic markers, glutamate release by microdialysis, and apoptosis in the hippocampus. Given that pyrethroids can affect inflammatory markers (Mizoguchi and Monji, 2017), we measured cytokines in the same brain regions implicated in the behavioral effects.

## 2 Materials and methods

### 2.1 Animals and treatment

Experiments were approved by the Institutional Animal Care and Use Committee of Cincinnati Children’s Research Foundation and adhered to guidelines on the care and use of animals in research by the U.S. National Institutes of Health. Male and nulliparous female Sprague Dawley rats (175–200 g upon arrival, CD IGS; strain No. 001, Charles River, Raleigh, NC) were acclimated for at least 7 days on an *ad libitum* NIH-07 diet (LabDiet, Richmond, Indiana) with reverse osmosis filtered/UV sterilized water. Rats were housed in a AAALAC International accredited vivarium. The housing room was maintained on a 14–10 h light–dark cycle (lights on at 600 h) with controlled temperature (20 ± 1 °C) and humidity (50 % ± 10 %). Females were paired with males in cages with hardwood chip bedding and stainless-steel enclosures for enrichment until females were pregnant, at which time females were housed separately (Vorhees et al., 2008). Day of birth was designated P0. On P3 pups were marked by subcutaneous injection of India ink. On P7 pups were ear punched for identification. Littermates were assigned to one of two groups (0 mg/kg corn oil; CO) or 1.0 mg/kg deltamethrin (DLM)) using a random number table. DLM (Bayer Crop Sciences, Frankfurt Germany, >99 % pure) was dissolved in corn oil (Arcos Organics, Geel, Belgium) in a dosing volume of 5 mL/kg and administered once per day to pups by gavage from P3-20 (Pitzer et al., 2019). We previously showed that a 5 mL/kg dosing volume of corn oil provides absorption with peak concentrations in P15 rats for at least 6 h post-treatment (Williams et al., 2019). This dosing volume was also used by others (Andrade et al., 2002, Mortuza et al., 2018). Litters were culled to 8 pups on P3 balancing for sex. Those testing the rats were blind to treatment group. Litters used for early assessments were euthanized 4 h following DLM, this is approximately when peak brain concentrations occur after an oral dose of DLM of 0.4–10 mg/kg (Mortuza et al., 2018, Williams et al., 2019). Others use an oral dosing volume of 0.2 mL/kg DLM in corn oil (Crofton and Reiter, 1984), 0.5 mL/kg (Anadón et al., 1996) or 1.0 mL/kg (Crofton and Reiter, 1988, Crofton et al., 1995, Godin et al., 2006, Hossain et al., 2019). We chose 5 mL/kg based on our previous studies (Pitzer et al., 2019, Williams et al., 2019) that showed behavioral effects using this procedure. Rats used for later assessments were weaned on P28 and housed 2/cage of the same sex. Offspring were weighed on P3 and daily thereafter until the end of dosing then weekly until the end of the experiment. Dams were weighed on P3 and weekly from P7 to weaning.

### 2.2 Early assessments (P21)

#### 2.2.1 Pro-caspase-3

Pro-caspase-3 expression in male and female rats was assayed after P3-20 DLM treatment in offspring from 12 litters with 1 rat/sex/group/litter on P21 by western blot. Antibodies were mouse anti-caspase-3 (31A1067: sc-56053, Santa Cruz Biotechnology, Inc, Cell Signaling Technology, Dallas, TX) at 1:150 with Odyssey IRDye 680 secondary antibody at 1:500 dilution and rabbit anti-β Actin (P/N: 926-42210, LI-COR Biosciences, Lincoln, NE) at 1:2,000 with Odyssey IRDye 800 secondary antibody at 1:15,000 dilution. Relative protein levels were quantified using the LI-COR Odyssey® scanner and Image Studio software for fluorescent intensity of each sample normalized to actin/lane and the gel normalized to highest actin sample.

#### 2.2.2 Cleaved Caspase-3

A separate group from 28 litters was used for western blot analysis of cleaved caspase-3 at different ages: P3, 9, 15, and 20. At each age whole hippocampus, neostriatum, and nucleus accumbens were dissected 4 h following DLM. Western blots were performed using Cell Signaling Technology’s western blot procedure (Cell Signaling Technology, Danvers, MA). Briefly, 25 µL of sample was mixed with Laemmli buffer (Sigma, St. Louis, MO), loaded on a 12 % gel (Bio-Rad Laboratories, Hercules, CA), and run at 200 V for 35 min in running buffer (25 mM Tris, 192 mM glycine, 0.1 % SDS). The gel was transferred to nitrocellulose transfer membrane (0.2 µm pore; Bio-Rad Laboratories, Hercules, CA) in 1X rapid transfer buffer at 40 V for 1.5 h. Membranes were first washed in 1X TBS for 5 min, then blocking buffer (1X TBS/5% dry milk) for 3 h, then incubated overnight at 4 °C with primary antibody in blocking buffer with 0.1 % Tween 20 (Sigma, St. Louis, MO). The following day membranes were washed in 1X TBST 3 times for 5 min each. Membranes were incubated with secondary antibody in blocking buffer with 0.1 % Tween 20 for 1 h at room temperature. Antibodies were rabbit anti-cleaved caspase-3 (Asp175; #9661, Cell Signaling Technology, Danvers, MA) at 1:200 with Odyssey IRDye 800 secondary antibody at 1:1,000 dilution and mouse anti-β actin (P/N: 926-42212, LI-COR Biosciences, Lincoln, NE) at 1:2,000 with Odyssey IRDye 680 secondary antibody at 1:15,000 dilution. Relative protein levels were quantified using the LI-COR Odyssey® scanner and Image Studio software. Inspection of the data distribution revealed that the dataset was not normally distributed, therefore, the data were log transformed prior to statistical analysis.

#### 2.2.3 Terminal deoxynucleotidyl transferase dUTP nick end labeling (TUNEL)

From 11 litters, P21 males were used to assess DNA fragmentation via TUNEL staining. Rats were administered 0.15–0.3 mL pentobarbital prior to a ventral incision and then a butterfly needle was inserted into the left ventricle to perfuse ice cold 1X PBS. Then, 30–60 mL of ice cold 4 % PFA in 1X PBS (pH 7.4) was perfused, and brains were removed and stored in 4 % PFA. The following day brains were transferred to 30 % sucrose in 1X PBS solution. After brains sank, sagittal sections (40 μM) were cut on a cryostat and mounted on slides. Slices were allowed to dry at room temperature overnight and stored at −80 **°**C. The TACS 2 Tdt-Blue Label *In Situ* Apoptosis Detection Kit (Trevigen, Gaithersburg, MD) was used for TUNEL assessment. Slides were equilibrated to room temperature for 2 h, rehydrated, and washed in 1X PBS twice for 10 min each. Tissue sections were permeabilized with 50 μL of Cytonin Solution for 30 min at 37 °C in a humidity chamber. Slides were washed twice in Milli-Q water (2 min each), immersed in quenching solution (45 mL methanol and 5 mL fresh 30 % hydrogen peroxide) for 5 min, washed in 1X PBS for 1 min and immersed in 1X Tdt labeling buffer for 5 min. Samples were removed from buffer and covered in 50 μL of labeling reaction mixture at 37 **°**C in a humidity chamber for 60 min then immersed in 1X Tdt stop buffer for 5 min. Slides were washed twice in 1X PBS for 5 min then covered with 50 μL of the Strep-HRP solution, placed in a humidity chamber and incubated at 37 **°**C for 10 min, and washed 2 times for 2 min each in 1X PBS. Slides were covered with 50 μL of Blue label solution for 5 min and washed twice in Milli-Q water for 2 min each. Samples were then counterstained by being placed in Milli-Q water and then in Nuclear Fast Red solution for 2 min each. Samples were dehydrated and cleared with xylene before mounting with Krystalon (Millipore, Burlington, MA). After hardening, sections were imaged, using a Nikon NiE upright Widefield microscope at 10X magnification under bright field illumination. Images were analyzed for TUNEL positive cells using RGB analysis on Nikon NIS-Elements AR analysis software (5.20.00 64-bit) in the striatum (neostriatum and nucleus accumbens were analyzed together as one image) and hippocampus (CA1-3 and dentate gyrus). The dentate gyrus was further analyzed via stereological counting of TUNEL positive cells. As a positive control, a subset of samples were treated with TAC-nuclease (Trevigen, Gaithersburg, MD) to confirm adequate permeabilization of membranes and staining quality.

#### 2.2.4 Cytokine assay

Thirteen separate litters were used to assess cytokines at P20 (1 male/group/litter was assessed). Cytokines were interferon gamma (IFN-γ), interleukin 10 (IL-10), interleukin 13 *(*IL*-*13*),* IL*-1β,* interleukin 4 *(*IL*-*4*),* interleukin 5 *(*IL-5), interleukin 6 (IL-6), keratinocyte chemoattractant/human growth-regulated oncogene (KC*/*GRO or CXCL1), and TNF-α. Hippocampus, nucleus accumbens, and striatum were dissected 4 h following the last dose on P20. Cytokines were assayed using the *Meso* Scale Discovery (MSD) proinflammatory panel-2 for rats V-PLEX® kit (MSD, Rockville, MD). Blocker H (150 μL; MSD, Rockville, MD) was added to each MSD 10-spot, 96 well plate and incubated for 1 h on a shaker at room temperature. The plate was washed 3 times with washer buffer (150 μL/well; 1X PBS + 0.05 % Tween 20), then samples and MSD calibrators were added to the plate, 50 μL/well. MSD calibrators were reconstituted by adding Diluent 42 to the highest calibrator, then serial dilutions were performed to create a set of calibrators, with Diluent 42 being the zero-calibrator containing no protein. Sample protein supernatants were also prepared with Diluent 42 (1:2 dilution). The plate was loaded with samples and calibrators, sealed, incubated, and placed on a shaker for 2 h. The plate was washed 3 times with wash buffer and 25 μL/well of 1X detection antibody solution added. The plate was sealed and incubated for 2 h on a shaker. The plate was washed 3 times with wash buffer, followed by 150 μL/well of a 2X read buffer, and the plate analyzed by an MSD *Meso* Sector S600 plate reader. Calculated concentrations were determined using MSD Discovery Workbench software®. Cytokine concentrations were normalized to total protein of each sample determined using BCA^TM^ Protein Assay Kit (Pierce Biotechnology, Rockford, IL). The assay was performed twice several months apart. Reference levels were different between assays therefore, the data from both assays were expressed as percent change from control before data were merged.

### 2.3 Adult assessments (P60)

#### 2.3.1 Long-term potentiation

Twelve litters with 4 males and 4 females (2/sex/treatment/litter) were used for LTP, with 1 rat/treatment/sex/litter assessed. At approximately P60, rats for LTP were decapitated, brains removed, sliced parasagittally at 350 μm using a vibratome, and placed on MED64 multielectrode arrays (Alpha Med Sciences, Kadoma, Japan) with an 8x8 array of contact electrodes (50x50 mm and spaced 150 mm apart) (Shimono et al., 2002). LTP was assessed in the CA1 region of the hippocampus (Pitzer et al., 2019). Slices were maintained in aCSF (saturated with 95 % O_2_ / 5 % CO_2_, at 32 °C). Recordings were followed until a stable baseline of field excitatory postsynaptic potentials (fEPSPs) were obtained (∼10 min), then a theta burst [tetanus = 100 Hz in 10 bursts (4 pulses/burst)] was delivered at a frequency of 5 Hz for 2 s and fEPSPs recorded for an additional 90 min (Amos-Kroohs et al., 2017, Pitzer et al., 2019). Data are reported as percent change from baseline.

#### 2.3.2 Western blots

A second pair from each group per litter was used for western blot analysis at approximately P60. Brain regions analyzed were hippocampus, striatum, and nucleus accumbens for DAT, DRD1, DRD2, *N*-methyl-d-aspartate (NMDA) receptor subunits NR1, NR2A, and NR2B, and α-amino-3-hydroxy-5-methyl-4-isoxazolepropionic acid (AMPA) receptors GluR1 and GluR2. Actin was the reference protein. Tissue was dissected, placed on dry ice, and stored at −80 °C. Frozen tissue was homogenized in immuno-precipitation assay buffer (25 mM Tris, 150 mM NaCl, 0.5 % sodium deoxycholate, and 1 % Triton X-100 adjusted to 7.2 pH) with protease inhibitor (Pierce Biotechnology, Rockford, IL). Protein was quantified using the BCA^TM^ Protein Assay Kit (Pierce Biotechnology, Rockford, IL) and samples were diluted to 3 µg/µL. Western blots were performed using LI-COR Odyssey® analyzer (LI-COR Biosciences, Lincoln, NE). Briefly, 25 µL of sample was mixed with Laemmli buffer (Sigma, St. Louis, MO) and loaded on 12 % gel (Bio-Rad Laboratories, Hercules, CA) and run at 200 V for 35 min in running buffer (25 mM Tris, 192 mM glycine, 0.1 % SDS). The gel was transferred to Immobilon-FL transfer membrane (Millipore, Burlington, MA) in 1X rapid transfer buffer (AMRESCO, Solon, OH) at 40 V for 1.5 h. Membranes were soaked in Odyssey PBS blocking buffer (LI-COR Biosciences, Lincoln, NE) for 1 h and incubated overnight at 4 °C with primary antibody in blocking buffer with 0.2 % Tween 20. Membranes were incubated with secondary antibody in blocking buffer with 0.2 % Tween 20 and 0.01 % SDS for 1 h at room temperature. Antibodies were rabbit anti-NMDA-NR1 (Ab109182, AbCam, Cambridge, MA) at 1:4,000 with Odyssey IRDye 800 secondary antibody at 1:3,000 dilution; rabbit anti-NMDA-NR2A (Ab124913, AbCam, Cambridge, MA) at 1:9,000 with Odyssey IRDye 800 secondary antibody at 1:20,000 dilution; rabbit anti-NMDA-NR2B (Ab81271, AbCam, Cambridge, MA) at 1:5,000 with Odyssey IRDye 800 secondary antibody at 1:20,000 dilution; rabbit anti-ionotropic glutamate receptor 1 (GluR1) (Ab109450, AbCam, Cambridge, MA) at 1:9,000 with Odyssey IRDye 800 secondary antibody at 1:15,000 dilution; rabbit anti-ionotropic glutamate receptor 2 (GluR2) (Ab133477, AbCam, Cambridge, MA) at 1:7,000 with Odyssey IRDye 800 secondary antibody at 1:10,000 dilution; rabbit anti-DRD1 (Ab40653, AbCam, Cambridge, MA) at 1:1,000 with Odyssey IRDye 800 secondary antibody at 1:3,000 dilution; rabbit anti-DRD2 (Ab85367, AbCam, Cambridge, MA) at 1:500 with Odyssey IRDye 800 secondary antibody at 1:3,000 dilution; rabbit anti-DAT (Ab184451, AbCam, Cambridge, MA) at 1:2,000 with Odyssey IRDye 800 secondary antibody at 1:20,000 dilution; and mouse anti-β Actin (P/N: 926-42212, LI-COR Biosciences, Lincoln, NE) at 1:2,000 with Odyssey IRDye 680 secondary antibody at 1:15,000 dilution. Relative protein levels were quantified using the LI-COR Odyssey® scanner and Image Studio software for fluorescent intensity of each sample normalized to actin.

#### 2.3.3 Hippocampal glutamate microdialysis

Another 11 litters were used for microdialysis using the same litter design as before except only males were used. Rats were ∼P60 when implanted with a concentric dialysis probe into the hippocampus such that the tip of the probe was located at the following coordinates: A/P, −3.6 mm relative to bregma; L, 2.0 mm; V, −3.8 mm (Paxinos et al., 1985). The surgical procedure was completed under isoflurane (2 %–4%; IsoThesia; Butler Animal Health Supply, Dublin, Ohio) anesthesia 72 h prior to testing. On the morning of dialysis, probes were connected to an infusion pump that delivered Dulbecco’s PBS (2 mL/min) for 3 h. A baseline sample was collected at 10, 20, 30, 60, 90, 120, and 150 min following administration of potassium (80 mM, dissolved in dialysis buffer) (Nair and Gudelsky, 2004). Dialysate glutamate was quantified by HPLC (Nair and Gudelsky, 2004). Placement of dialysis probes was verified post-mortem in coronal sections.

### 2.4 Data analysis

Data were analyzed using mixed linear model ANOVAs (Proc Mixed, SAS v9.4, SAS Institute, Cary, NC). Fixed factors were treatment and sex in most cases. Interval or time was the repeated measure (RM) in RM-ANOVA models for LTP and microdialysis. For these an autoregressive-1 covariance structure was used. RM-ANOVA was also used for TUNEL for dentate gyrus cell counts. To control for litter, only one rat per treatment per sex per litter was used for any outcome and in these analyses litter was a random factor in ANOVA models with first-order Kenward-Roger degrees of freedom. Significant interactions were further analyzed using the SAS slice option in Proc Mixed, which is an ANOVA at each level of a variable (usually the repeated measure dimension) that uses the MSE (mean square error) from the overall ANOVA for each comparison (see SAS v9.4 Manual). Treatment by sex ANOVAs were conducted for LTP and westerns. Because the two cytokine assays had different baselines, the data were combined as percent of control and square root transformed prior to analysis. Data are presented as least square (LS) mean ± SEM. Statistical significance was set at p ≤ 0.05.

## 3 Results and discussion

### 3.1 Apoptosis

Caspase-3 is a proenzyme that activates cell death when cleaved by an initiator caspase. Cleaved caspase-3 then activates caspase-9 to trigger apoptosis (Salvesen, 2002, Ghavami et al., 2009, Walters et al., 2009). DLM triggers caspase cascades by causing ER stress that leads to unfolded protein protective mechanisms and apoptosis, both in cell culture and *in vivo* in adult mice (Hossain and Richardson, 2011, Hossain et al., 2015, Hossain et al., 2019). We examined cleaved caspase-3 expression on P3, P9, P15, and P20 and pro-caspase-3 on P21 in hippocampus and striatum in males. No significant differences were found in either the hippocampus or striatum for pro- or cleaved caspase-3 (Figure S1 and S2). However, caspases are difficult to measure in the preweaning period (Mooney and Miller, 2000), and this may have limited our ability to detect effects. Because of this we turned to TUNEL staining.

**Fig. 1:**
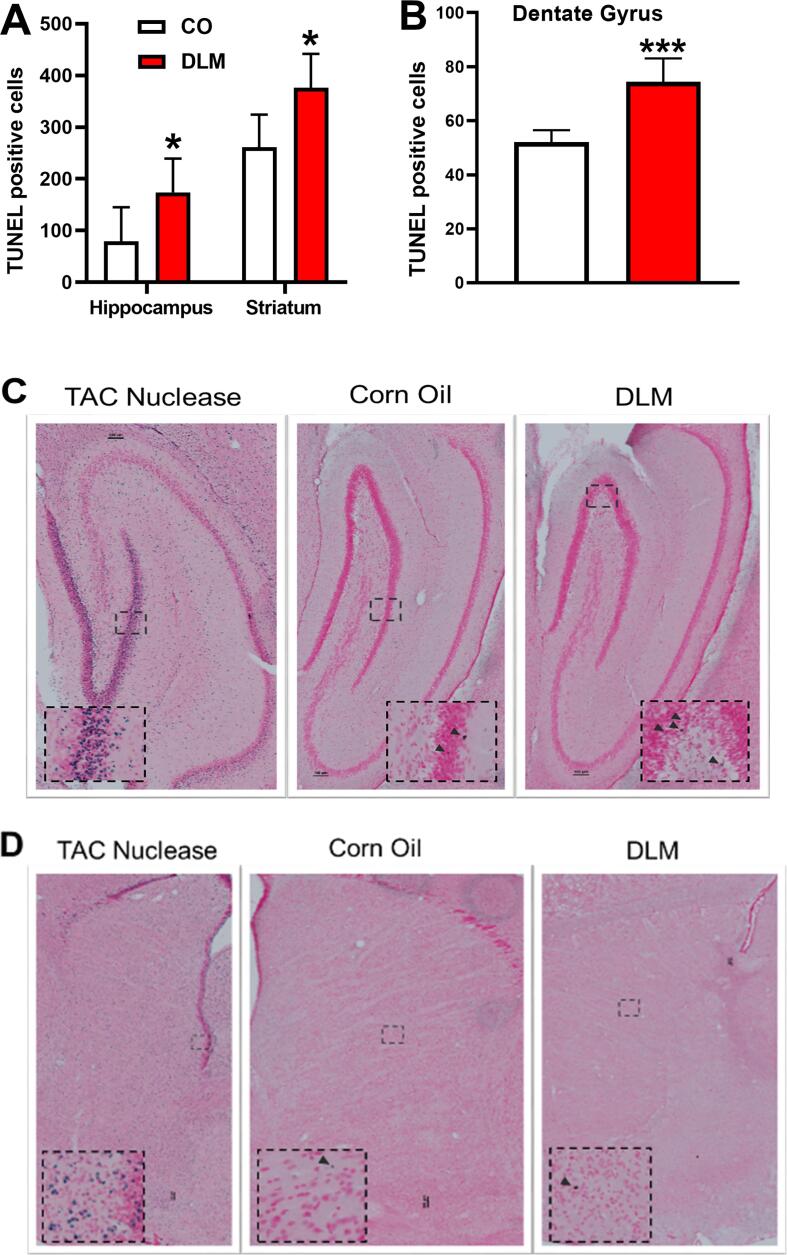
TUNEL Immunohistochemistry in striatum and dorsal hippocampus at P21. (A) Quantification of spot detection in the striatum and hippocampus for TUNNEL-positive cells in corn oil controls and DLM treated P21 males rats, (B) Stereological counts of TUNEL-positive cells in the dentate gyrus of DLM and control P21 male rats. Images are of the hippocampus (C) and striatum (D) for TAC nuclease (positive control) from corn oilcontrols and DLM treated rats, respectively (left to right). Inset is an enlarged image shown in dashed box. Scale = 100 mm. Black arrows are TUNEL positive cells shown in inset. Sample size: n = 8–11/treatment/region. *p < 0.05; ****p < 0.0001 compared with corn oil control. DLM = deltamethrin.

TUNEL staining showed that DLM increased cell death in the hippocampus and neostriatum compared with controls. There was an effect of treatment [F(1, 21.8) = 4.28, p < 0.05], with increased TUNEL positive cells in both regions (Fig. 1**A, C and D**) compared with controls. We also observed an effect of region [F(1, 20.9) = 15.23, p < 0.001], with higher counts for TUNEL positive cells in the neostriatum.

Increases in TUNEL positive cells, were also observed by others following a single dose of DLM (6 mg/kg; gavage) in adult male mice, mediated by caspase cascades, as seen by increased levels of caspase-12 and caspase-3 (Hossain et al., 2019). The latter could be blocked by inhibiting ER stress activation through administration of salubrinal (1 mg/kg i.p.; 24 h and 30 min before DLM administration), a eukaryotic translation initiating factor 2 subunit 1 (eIf2α) inhibitor (Hossain et al., 2019). Differences in dose, age, and species may account for differences between what Hossain et al. observed vs our data with regard to caspase activity. This warrants further investigation. Additionally, DLM may induce cell death through mechanisms other than caspase-3 that might account for these differences. For example, cell death may also occur through p53 signaling or oxidative stress (Kumar et al., 2015).

We examined TUNEL histochemistry in greater detail in the dentate gyrus region of the hippocampus using stereological counts (Fig. 1**B**). In this region, DLM-treated rats had increased numbers of TUNEL positive cells compared with controls [t(20) = −4.58, p < 0.001]. This is consistent with data that 5-bromo-2-deoxyuridine (BrdU) labeled cells, a marker for cell proliferation, are reduced in adult mice exposed to DLM (Hossain et al., 2015). The dentate gyrus is important because it is a region that generates new granule cells during the preweanling period (Bayer and Altman, 1974), that are thought to play a role in learning and memory and is a time period when we dosed DLM.

### 3.2 Cytokines

Cytokines are secreted by immune cells to initiate protective mechanisms in response to injury, infection, or toxic substances (Zhang and An, 2007, Turner et al., 2014), and some have been examined after DLM exposure. One study reported that DLM increased TNF-α levels in microglia cell culture (Hossain, 2016), indicating potential disruption of cytokines following DLM exposure. We assayed males at P20 by a multiplex assay for 9 cytokines. We found one significant effect (KC/GRO) and three trends in different brain regions (Table 1).

**Table 1:** Cytokine analysis: Cytokines were assessed in control and DLM-treated male rats at P20 in neostriatum, nucleus accumbens, and hippocampus. Data were normalized for concentration (pg/mL). Sample size: n = 6–13/treatment/region. †p < 0.10; ***p < 0.001 compared with control. Abbreviations: IFN- γ = interferon gamma; IL*-1β,* interleukin 1-beta; IL*-*4, interleukin-4*;* IL-5, interleukin-5*;* IL-6*,* interleukin-6; IL-10, interleukin-10; IL*-*13, interleukin-13*;* KC/GRO (CXCL1), keratinocyte chemoattractant/human growth-regulated oncogene; TNF-α, tumor necrosis factor-alpha.

	Striatum		Nucleus Accumbens		Dorsal Hippocampus	
	**CO**	**DLM**	**CO**	**DLM**	**CO**	**DLM**
Cytokine						
INF-γ	100 ± 11.2	128.7 ± 11.7†	100 ± 11.2	100.3 ± 11.7	100 ± 11.2	123.2 ± 11.7
IL-10	100 ± 16.2	105.6 ± 16.3	100 ± 11.4	112.0 ± 11.9	100 ± 11.1	101.4 ± 12.4
IL-13	100 ± 22.1	120.3 ± 23.0	100 ± 22.1	104.8 ± 23.0	99.7 ± 23.1	138.8 ± 23.0†
IL-1 β	100 ± 9.3	83.8 ± 9.7	100 ± 9.3	75.3 ± 9.7†	100.0 ± 9.3	96.7 ± 9.7
IL-4	100 ± 20.6	91.3 ± 20.6	100 ± 13.8	99.5 ± 14.3	99.9 ± 13.8	122.1 ± 15.0
IL-5	100 ± 7.4	109.3 ± 7.7	100 ± 7.4	103.1 ± 7.7	100.0 ± 7.4	114.8 ± 7.7
IL-6	100 ± 10.9	105.1 ± 11.4	100 ± 11.0	74.4 ± 11.4	100.0 ± 10.9	108.4 ± 11.4
KC/GRO (CXCL1)	99.9 ± 18.3	101.2 ± 19.1	100 ± 18.3	90.4 19.1	99.9 ± 18.3	192.6 ± 19.1**
TNF-α	99.9 ± 12.4	86.9 ± 12.9	100 ± 12.4	96.0 ± 12.9	100.0 ± 12.4	96.0 ± 12.9

Two multiplex assays were run. Within each assay samples were consistent but there was a difference across assays, therefore, values were standardized to control levels and square root transformed for statistical analysis by ANOVA.

^†^p < 0.10, ***p < 0.01 compared with CO.

IFN-γ is an immune regulating cytokine and when present in the central nervous system it can cause pathological changes, such as alterations in myelination, gliosis, and disruption of cell migration (Popko et al., 1997), which could contribute to changes in behavior. The effects of DLM on IFN-γ are largely unknown, with one study reporting that DLM reduced IFN-γ in adult rat thymocytes (Kumar et al., 2014). We saw a trend in which IFN-γ was modestly increased in the striatum (F(1, 67.46) = 3.43, p = 0.069) (Table 1).

IL*-*1β is increased in the hippocampus of adult male mice exposed to DLM (Hossain et al., 2022). We found a trend toward IL-1β being decreased in the nucleus accumbens (F(1, 67.39) = 3.63, p = 0.061). IL*-*1β is a proinflammatory cytokine that has modulatory effects beyond the immune system. Disrupted IL-1β signaling can impair memory (Barrientos et al., 2002, Hein et al., 2007) and cell survival (Albrecht et al., 2002, John et al., 2005, Saavedra et al., 2007, Hewett et al., 2012), which perhaps is involved in the learning and memory changes we observed in the Cincinnati water maze (CWM) (Pitzer et al., 2019).

KC/GRO (CXCL1) was significantly increased in the hippocampus (F(1,65.64) = 8.20, p < 0.01) (Table 1). No effects were seen in the striatum or nucleus accumbens. KC/CRO directs neutrophils to injured tissues (Shaftel et al., 2007) which are critical for maintaining tissue homeostasis and pathogen elimination and plays a role in neuroinflammatory disorders. At pathological levels, neutrophils acquire a toxic phenotype when transmigrating to the central nervous system (Rossi et al., 2020). Neutrophils contribute to the pathogenesis of Alzheimer’s disease, including the associated cognitive impairments (Zenaro et al., 2015). This suggests that cytokine changes in DLM-exposed rats may contribute to behavioral changes. The observed KC/GRO effect in the hippocampus could have implications for changes in hippocampal dependent learning and memory in the MWM (Pitzer et al., 2019).

We also observed an increased trend in IL-13 [F(1, 67.67) = 2.99, p = 0.088] in the hippocampus of DLM-exposed rats. IL-13 can be neuroprotective (Mori et al., 2016) or proinflammatory (Brombacher et al., 2017).

Taken together the brain cytokine changes found in DLM-treated rats could indicate compromised immune responses. Examining additional timepoints during or after our exposure period may also clarify these preliminary observations as would including females in follow up experiments, since females have heightened immune responses compared with males (Klein and Flanagan, 2016).

### 3.3 Dopamine

**Fig. 2:**
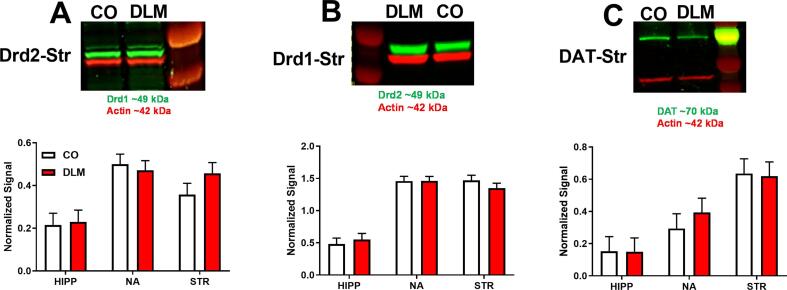
Dopamine receptor and transporter levels by western blots: (A) DRD1, (B) DRD2, (C) DAT protein expression in hippocampus, nucleus accumbens, and striatum after DLM or CO. Data are averaged normalized signal relative to actin. Target protein is labeled in green and actin in red. Sample size: n = 7–10/treatment/sex/region. DA, dopamine; DRD1, dopamine D1 receptor; DRD2, dopamine D2 receptor; DAT, dopamine transporter; HIPP, hippocampus; NA, nucleus accumbens; STR, striatum. (For interpretation of the references to colour in this figure legend, the reader is referred to the web version of this article.)

Previously, DLM-exposed rats showed deficits on egocentric navigation in the CWM (Pitzer et al., 2019). The CWM is a striatally based form of learning largely mediated by DA (Braun et al., 2015, Braun et al., 2016, Vorhees and Williams, 2016). Consistent with these deficits, *Drd1* mRNA in the neostriatum and DA stimulated release in the nucleus accumbens were decreased in DLM-treated rats (Pitzer et al., 2019). However, in the current experiment, western blots showed no significant effects of DLM treatment on DRD1, DRD2, or DAT in the offspring as adults (Fig. 2).

Although, the CWM involves striatal DA (Braun et al., 2015, Braun et al., 2016, Vorhees and Williams, 2016), the absence of effects on levels of DRD1 and DRD2 after DLM suggest that these receptors are not likely to be involved in the CWM learning deficits. How decreased *Drd1* mRNA and reduced stimulated DA release contribute to the CWM changes is unknown.

We did not find the changes in DA as seen in a developmental mouse model of DLM exposure (Richardson et al., 2015). Richardson et al. (2015) found increased DAT levels in striatum in males which we did not see, but we both found attenuated DA release in the nucleus accumbens. Richardson et al. (2015) also found increased DA receptor density in the striatum using radioligand binding assays for DRD1 and DRD2. DRD1 was increased only in males, in females DRD1 was unchanged as was DRD2. We assessed DRD2 and DAT in striatum by western blot and found no differences (Pitzer et al., 2019), but this may be due to differences in methods. Richardson et al. (2015) administered DLM once every-three days throughout gestation and lactation, whereas we administered DLM daily during lactation only. Yet both studies find changes in DA in the striatum and/or nucleus accumbens. There are many experimental differences that may account for why the mouse and rat outcomes differ, including different species, exposure periods, DLM doses, dose spacing, delivery methods, handling, and environmental conditions.

### 3.4 Glutamate

**Fig. 3:**
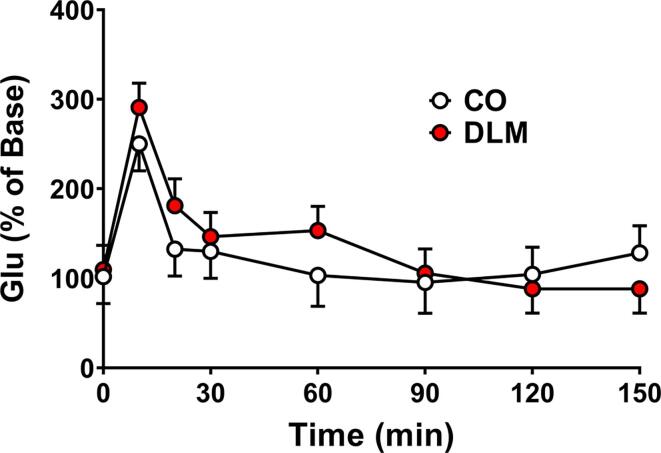
Extracellular glutamate release: Potassium (80 mM) stimulated glutamate release by microdialysis in dorsal hippocampus of male DLM and CO treated rats. Data are percent of baseline (time zero) over a 150 min sample collection period. Sample size: n = 6/treatment.

To explore the effects of the altered LTP and cognitive flexibility deficit following developmental DLM exposure several glutamatergic markers, i.e., AMPA and NMDA receptors, were assessed (Williams et al., 2007, Luscher and Malenka, 2012, Nicoll, 2017) as well as glutamate release by *in vivo* microdialysis. There were no effects found on potassium stimulated hippocampal glutamate release (Fig. 3). There was a main effect of time [F(7, 62.9) = 11.62, p < 0.0001], with glutamate release increasing for 10 min following stimulation and then returning to baseline as expected.

**Fig. 4:**
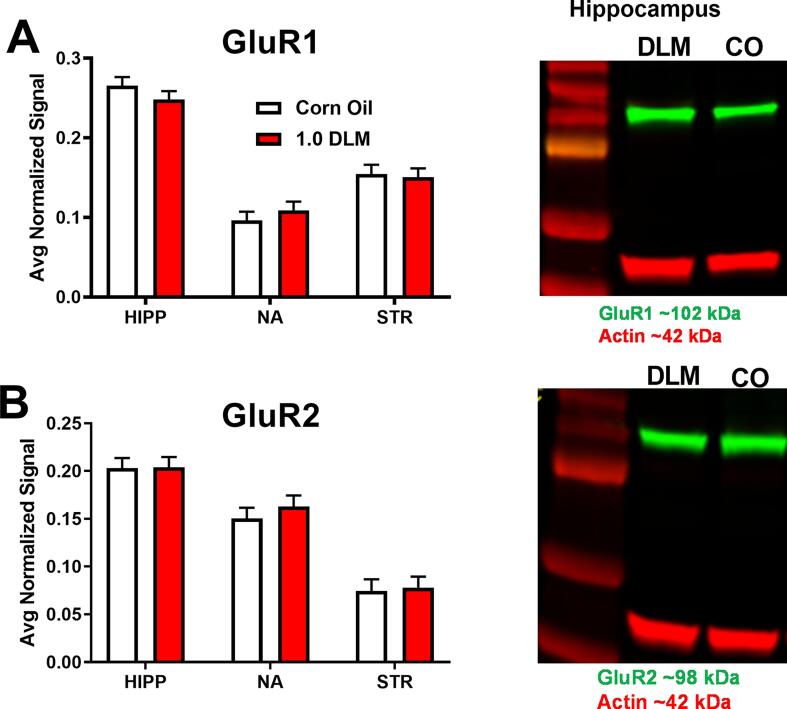
AMPA receptor subunit western blots: (A) GLUR1, (B) GLUR2 protein expression in hippocampus, nucleus accumbens, and striatum. Data are normalized to actin. GLUR1 (∼102 kDa) and GLUR2 (∼98 kDa) are in green and actin (∼42 kDa) in red. Sample size: n = 7–10/treatment/sex/region. Abbreviations: GLUR1, AMPA receptor subunit R1; GLUR2, AMPA receptor subunit R2; HIPP, hippocampus; NA, nucleus accumbens; STR, striatum. (For interpretation of the references to colour in this figure legend, the reader is referred to the web version of this article.)

**Fig. 5:**
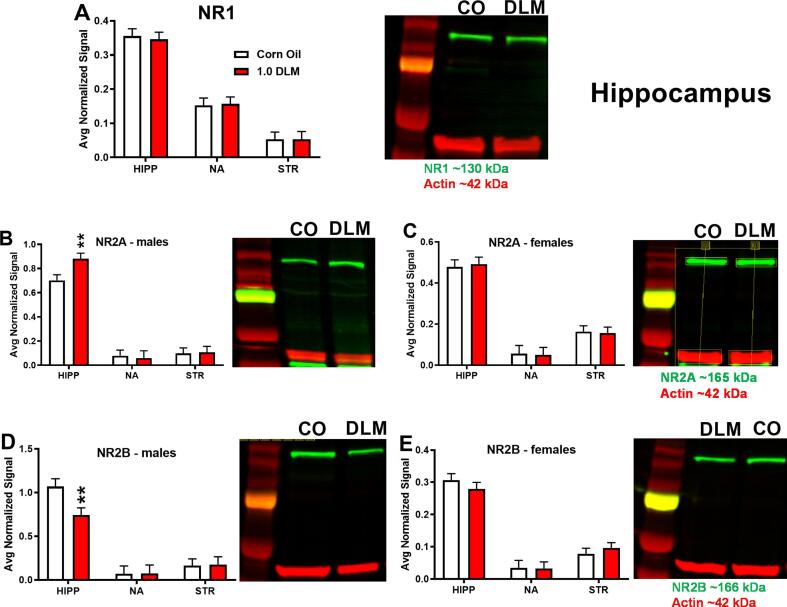
NMDA receptor subunit western blots: (A) NMDA-NR1 in hippocampus, nucleus accumbens, and striatum. (B) NMDA-NR2A in the same regions for males. (C) NMDA-NR2A for females. (D) NMDA-NR2B for males. (E) NMDA-NR2B for females. Data are normalized to actin. NR1 (∼130 kDa), NR2A (∼165 kDa), and NR2B (∼166 kDa) are in green, and actin (∼42 kDa) in red. Sample size: n = 7–10/treatment/sex/region. **p < 0.01 compared with CO. Abbreviations: HIPP, hippocampus; NA, nucleus accumbens; STR, striatum. (For interpretation of the references to colour in this figure legend, the reader is referred to the web version of this article.)

There were no significant DLM associated changes on AMPA receptors GluR1 (Fig. 4**A**) or GluR2 (Fig. 4**B**) nor effects on NMDA-NR1 levels (Fig. 5**A**). For NMDA-NR2a there was a region × sex effect [F(2,112) = 75.22, p < 0.0001], therefore, we analyzed the data further by sex. In males, NMDA-NR2A levels showed an effect of region [F(2, 415) = 146.97, p < 0.0001], a trend for treatment [F(1, 18.8) = 2.97, p = 0.10], and trend for the treatment × region interaction [F(2, 34.7) = 2.67, p = 0.083]. Follow-up analyses showed that DLM-treated males had increased NMDA-NR2A levels in the hippocampus (Fig. 5**B**; p < 0.01) compared with controls. For NMDA-NR2B, there was also a region × sex effect [F(2,105) = 56.27, p < 0.0001], therefore, we analyzed these data by sex. For males, there was an effect of region [F(2, 40.1) = 56.3, p < 0.0001] and a treatment × region interaction trend [F(2, 40.1) = 2.59, p < 0.09] (Fig. 5**D**). The male DLM rats had decreased levels of NMDA-NR2B in the hippocampus (p < 0.01) compared with controls. There were no effects in females (Fig. 5**C,E**).

NMDA receptors are heteromers (dimers or trimers), composed of two NR1 subunits and two NR2 subunits (NR2A, NR2B, NR2C) or NR3 subunits (NR3A, NR3B) (Low and Wee, 2010, Flores-Soto et al., 2012, Vyklicky et al., 2014). NMDA-NR2A and -NR2B are present throughout the brain, including in areas involved in CWM and MWM learning. NR2A and NR2B undergo a developmental switch in expression (Wenzel et al., 1997). This occurs prior to weaning, during the same developmental window when we treated rats with DLM. It is possible that exposure to DLM during this period altered the transition of NMDA receptor subunits resulting in long-term changes in the composition of NMDA receptors. Increased NMDA-NR2A subunit ratios are associated with long-term depression (LTD) whereas increased NMDA-NR2B subunit ratios are associated with LTP changes (Yashiro and Philpot, 2008). LTD activity in DLM-treated offspring should be assessed in future experiments.

### 3.5 Long-Term potentiation

**Fig. 6:**
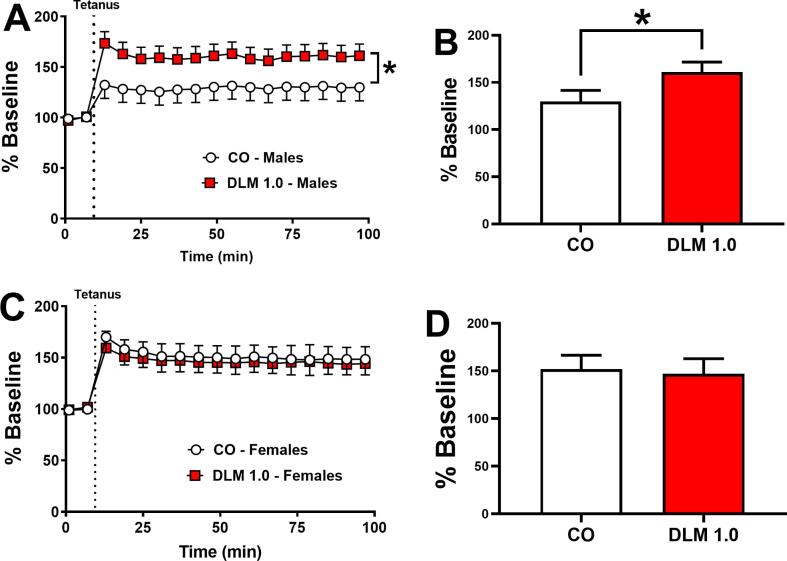
Dorsal hippocampal (CA1) LTP. (A) Excitatory postsynaptic field potentials (EPSP) over 90 min in males. (B) Male fEPSPs over 90 min. (C) Female fEPSPs over 90 min. (D). Female EPSPs averaged across min. Data are percent of baseline. Dotted lines show when the tetanizing stimulus was applied; tetanus = 100 Hz in 10 bursts [4 pulses/burst] delivered at a frequency of 5 Hz for 2 s after 10 min of stable baseline with EPSPs recorded for 90 min following stimulation. CO: n = 17 (9 males, 8 females); DLM: n = 17 (9 males, 8 females). *p < 0.05 compared with CO.

LTP increases in CA1 of the hippocampus were seen in P25-35 DLM-treated rats (Pitzer et al., 2019). To determine if this effect persists, offspring treated as before with DLM were tested in adulthood. The main effects of treatment: [F(1, 7.17) = 10.54, p < 0.05] and interval [F(89, 977) = 4.2, p < 0.0001] were significant for males (Fig. 6**A,B**), with DLM-treated rats showing increased LTP. No treatment effects were observed for females (Fig. 6**C,D**). No group differences were found prior to LTP induction, differences only appeared following tetanus.

Reduced LTP is typically associated with impaired spatial learning and memory, whereas we found increased LTP, similar to what we found before in younger rats (Pitzer et al., 2019). Moreover, the effect was sex-specific, affecting only males. Consistent with this, we observed deficits in the MWM only on reversal and shift trials in males (Pitzer et al., 2019). LTP is a cellular correlate of spatial learning and memory in the MWM (Morris et al., 1986, Bliss and Collingridge, 1993, Bannerman et al., 1995, Moser et al., 1998, Herring and Nicoll, 2016, Nicoll, 2017) although there are exceptions (Meiri et al., 1998, Garcia-Alvarez et al., 2015, Aziz et al., 2019). Impairments in MWM learning, while indicative of hippocampal dysfunction, are not always reflected by changes in LTP (Morris et al., 1982). We chose the CA1 region because it is critical for spatial learning and memory (Tsien et al., 1996, Oh et al., 2003, Suthana et al., 2009, Bannerman et al., 2014). There are knockout models in which altered behavior and/or learning and memory occur in conjunction with increased LTP (Jia et al., 1996, Migaud et al., 1998, Gu et al., 2002, Rutten et al., 2008).

Cross species comparisons of exposure suggest that the doses used in rodents are higher than those found in humans. We showed that in P15 rats, a 1 mg/kg oral dose of DLM in 5 mL/kg corn oil resulted in DLM concentrations of 100–200 μg/mL in plasma 2–6 h post-exposure (Williams et al., 2019). There are no comparable data in humans but extrapolating from urinary metabolites (*cis*-DCBA), intake of DLM is estimated to be ∼20.4 ng/kg with maximum intakes of ∼1.4 μg/kg (Klimowska et al., 2020). The NOAEL for humans is 1 mg/kg. The acceptable daily intake for DLM is 0–0.01 mg/kg/day (WHO, 2005), hence humans are exposed to levels lower than what is given to rodents. However, the purpose of toxicological studies is to identify hazards not necessarily to use doses to which humans are exposed.

## 4 Conclusions

The data presented here add evidence that preweaning DLM exposure affects glutamatergic systems (NMDA-NR2A and –NR2B receptors) and CA1 LTP. We observed increased apoptosis in DLM-treated rats that may affect neurogenesis in the dentate gyrus, a region critical for spatial learning. Future studies are needed to narrow the search for molecular changes from early DLM exposure. Experiments on cell death and inflammatory mediators may be informative given the trends we found in four cytokines. Overall, the data show multiple effects from developmental DLM exposure. These changes likely contribute to the learning and memory deficits found following developmental DLM exposure. Studies are needed to better understand how DLM changes to NMDA receptors affect LTP and learning.

## Animal welfare

This research was conducted in accordance with the U.S. National Research Council Guide for the Care and Use of Laboratory Animals.

## Declaration of Competing Interest

The authors declare that they have no known competing financial interests or personal relationships that could have appeared to influence the work reported in this paper.

## Data Availability

Data will be made available on request.
