# Supplementary material for: Developmental deltamethrin: Sex-specific hippocampal effects in Sprague Dawley rats
Source: Curr Res Toxicol. 2022 Nov 2;3:100093. doi: 10.1016/j.crtox.2022.100093 (PMC9661443; doi:10.1016/j.crtox.2022.100093)
Supplement: Supplementary data 1 [file mmc1.docx]

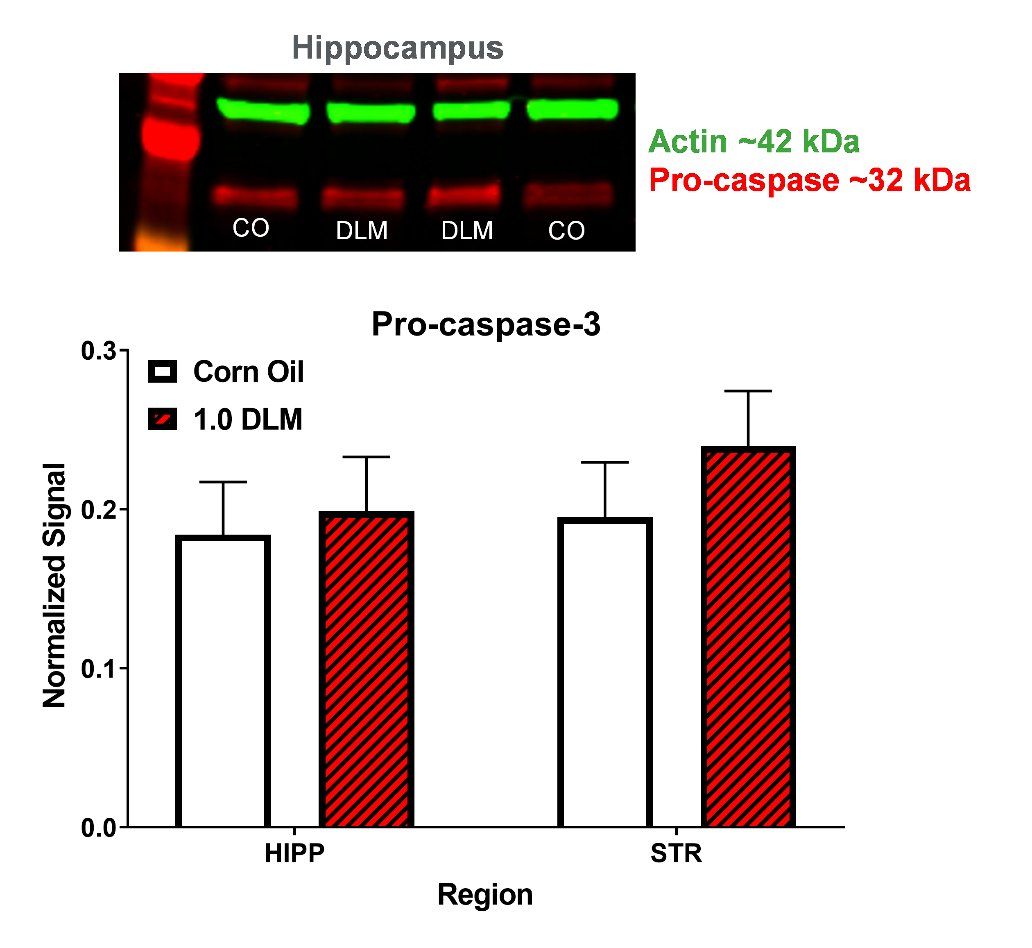


**Figure S1**. Pro-caspase-3 western blots: Protein expression in male rats treated with CO or DLM in hippocampus and striatum on P21. Representative blots with Pro-caspase-3 in red and actin in green. Sample size: n = 7-11/sex/treatment/region. Abbreviations: CO = corn oil; DLM = deltamethrin; HIPP = hippocampus; STR = striatum.


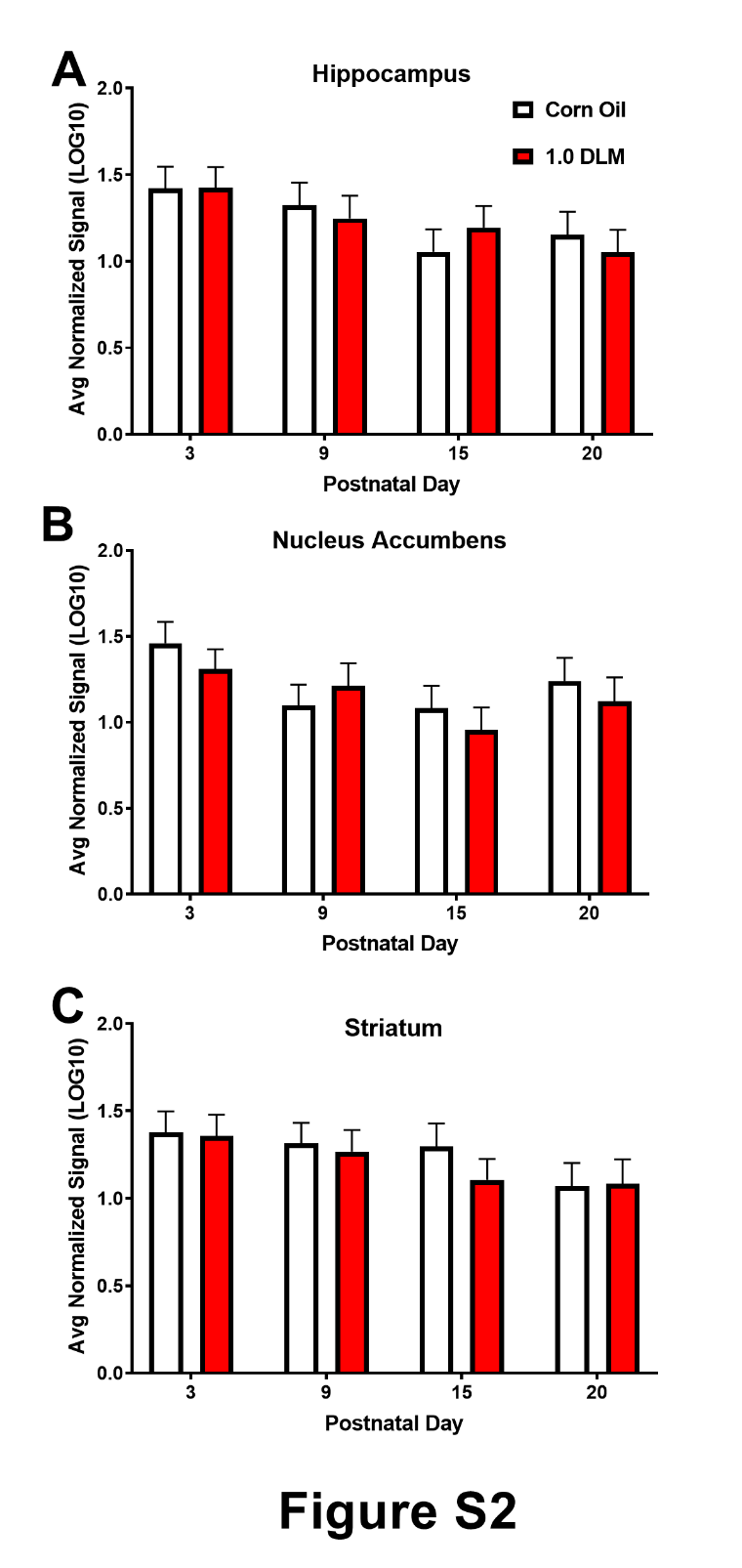


**Figure S2**. Cleaved Caspase-3 western blots in male rats treated with DLM or CO in (A) hippocampus (B), nucleus accumbens, and (C) striatum at P3, 9, 15, and 20. Cleaved caspase-3 is ~19/17 kDa vs. actin which is ~42 kDa. Sample size: n = 7-11/treatment/region. Abbreviations: CO = corn oil; DLM = deltamethrin; HIPP = hippocampus; NA = nucleus accumbens; STR = striatum; P = postnatal day.
